# A follow-up program in patients after hospitalization for heart failure: long-term health related quality of life and associated factors

**DOI:** 10.3389/fcvm.2024.1358390

**Published:** 2024-04-05

**Authors:** R. Paleckiene, D. Zaliaduonyte, V. Dambrauskiene, J. Macijauskiene

**Affiliations:** ^1^ Department of Cardiology, Lithuanian University of Health Sciences, Kaunas, Lithuania; ^2^ Nursing Management Service, Kaunas Hospital of the Lithuanian University of Health Sciences, Kaunas, Lithuania; ^3^ Department of Geriatrics, Lithuanian University of Health Sciences, Kaunas, Lithuania

**Keywords:** heart failure, monitoring program, health-related quality of life, outcomes, self-care

## Abstract

**Background:**

The well-being of individuals with chronic heart failure (HF) is significantly influenced by their health-related quality of life (HRQoL), which serves as a crucial measure indicating how HF affects their daily activities. Monitoring programs aimed at reducing the number of hospitalizations and improving functional conditions are currently being offered to patients with chronic HF.

**The objective:**

To examine the long-term health-related quality of life changes in patients with heart failure enrolled in a follow-up program after hospitalization and to evaluate the factors associated with quality of life of patients with heart failure.

**Methods:**

This prospective study was conducted between 2019 and 2020 at the Department of Cardiology of Lithuanian University of Health Sciences. Patients were divided into two groups: Group I consisted of 71 patients (60.2%) where the Minnesota Living with Heart Failure Questionnaire (MLHFQ) score decreased by more than 10 points at 4th visit if compared to the 1st one; and Group II consisted of 47 patients (39.8%) where the MLHFQ score remained unchanged or increased by less than 10 points at the 4th visit if compared to the 1st visit.

**Results:**

Statistically significant differences were observed between groups. In Group II, a history of myocardial infarction was more frequent (*p* = 0.038), and there was a significantly higher occurrence of significant coronary artery disease (*p* = 0.006). Laboratory parameters indicating liver function exhibited statistically significant deterioration among patients in Group II. Specifically, AST (*p* = 0.050), ALT (*p* = 0.010), and GGT (*p* = 0.031) levels significantly increased. Upon analyzing the echocardiographic data, a statistically significant difference was found between the groups in relation to the left ventricular ejection fraction (LVEF) (*p* = 0.043) and TAPSE (*p* = 0.031). An analysis of changes in dimensions related to QoL was conducted during the long-term follow-up program, which revealed statistically significant differences between groups in overall changes based on the MLHFQ (*p* < 0.001). This difference was also observed across all dimensions, including the emotional, physical, and social aspects (*p* < 0.001).

**Conclusion:**

Patients who had a higher LVEF at baseline, as well as those with an etiology of ischemic heart disease (IHD), better liver function, and fewer manifestations of edema, demonstrated a statistically significant improvement in their quality of life throughout the course of the patient monitoring program.

## 1 Introduction

Heart failure (HF) is among the chronic conditions that elevate the risk of mortality associated with cardiovascular diseases. HF is characterized as a syndrome involving symptomatic ventricular dysfunction that exhibits continuous progression (1). HF is now recognized as a worldwide pandemic, impacting over 26 million individuals globally, with 15 million of them residing in Europe. In 2020, data from the Institute of Hygiene indicated that more than 132.3 thousand patients with HF were documented in Lithuania. Consequently, nearly 47 out of 1,000 Lithuanian residents grapple with HF symptoms that disrupt their daily activities, thereby diminishing their quality of life (2). As HF advances or experiences decompensation, complications such as lung, kidney, and liver failure emerge, along with the onset of sleep apnea syndrome and anemia. These complications have adverse effects not only on the patient's physical and functional well-being but also on their HRQoL (3). Monitoring programs with the goal of decreasing hospitalizations among HF patients and enhancing their functional status and HRQoL are actively being developed and implemented globally (4, 5). Educational interventions focused on enhancing self-care skills in patients with chronic diseases, particularly those with cardiovascular conditions, can play a role in improving their overall health (6). Patients experiencing HF will encounter alterations in their care requirements due to the implications of the illness and its treatment. Effectively addressing the challenges associated with the disease necessitates a comprehensive understanding of self-care behaviors. The aspect of adherence to self-care behaviors is particularly crucial in these patients, as acquiring skills in self-care directly influences their comfort, functional capabilities, and the progression of the disease (7). Assessing the scale of the problem in 2015, training sessions for heart failure patients were initiated in Lithuania to help individuals to obtain necessary skills in self-care techniques. In the same year, the order by the Ministry of Health “On the consultation of a cardiologist and nurse, including patient education, provided to individuals with heart failure’ was issued, specifying participants and consultants of this program. It was determined that the cardiologists holding a valid cardiologist license and having a certificate of 72 h informal education program on “Heart Failure Diagnosis, Treatment, and Outpatient Care Organization” can be involved in the program. Nurses providing services in this program must hold a valid general practice nurse license and have completed a program of no less than 288 h titled “Specialized Care for Heart Failure Patients.” The two centers where specialists are trained are located in Lithuanian University of Health Sciences (Kaunas) and Vilnius University (Vilnius). In Lithuania, this is the only long-term monitoring and training program designed for patients with heart failure.

Hence, the aim of the current study was to assess the influence of a prolonged monitoring program on changes in HRQoL and clinical parameters among patients with HF after HF decompensation.

## 2 Methods and materials

### 2.1 Study population

In the years 2019 March–2020 December, an observational research study was carried out at the Department of Cardiology, Lithuanian University of Health Sciences. Data from the prospective cohort of 118 HF patients who were discharged from the Department of Cardiology at Lithuanian University of Health Sciences following the decompensation of HF, coded as I50 according to the International Classification of Diseases (ICD-10), chronic decompensated and *de novo* instances were subjected to analysis. Based on the order of the Minister of Health of the Republic of Lithuania No V-1330 of 24 November 2015 “On the approval of the requirements for the provision of a consultation by a cardiologist and a nurse, including patient education, to persons with heart failure” all patients had completed four consecutive training consultations of 1 year. All data was collected during consultations.

#### 2.1.1 Inclusion and exclusion criteria

The study included 118 patients who had four consultations with cardiologists and heart failure nurses within 12 months and were discharged from the hospital after treatment for acute HF or decompensated HF with reduced ejection fraction (EF). The patients included in the study provided written consent for the use of their personalized data for scientific purposes and for the publication of the obtained results publicly, ensuring their anonymity.

Exclusion criteria: Patients who did not complete the entire training and counseling program, life expectancy less than 1 year. or patients died within 1 year after HHF or unable to perform or well corporate with score assessment such as severe cognitive impairment or bed ridden status.

For a more thorough analysis, the participants were divided into two groups: Group I comprised 71 patients (60.2%) in whom the MLHFQ score decreased by more than 10 points (in accordance with the established standards of provision and quality of service for cardiologist and nurse consultation, including patient education, for individuals with heart failure, as per Order No. V-1330 of November 2015) at the fourth visit. Group II comprised 47 patients (39.8%) in whom the MLHFQ score either remained unchanged or increased by less than 10 points at the fourth visit. The patients received guideline-directed pharmacological and nonpharmacological treatments. During each visit, a cardiologic examination of the patient and laboratory tests were performed.

Four meetings with a nurse and a cardiologist were reserved for this training with the focus on self-care.

The training modules were organized according to the following topics:

Module 1: General knowledge of HF (concept, affects on other organs, clinical symptoms);
Module 2: Lifestyle limitations with HF (diet, physical activity, sex life);
Module 3: Treatment for HF (medications, electrical devices, surgical treatment, palliative treatment);
Module 4: Self-care in HF (nursing problem solving options, appropriate implementation of treatment and care plan, symptoms indicating deterioration).

Since the meetings are not held in groups, but in person, the confidentiality of a patient is ensured and opportunities to ask questions are provided.

The time of a consultation is shared by a cardiologist (30 min) and a nurse (1 h 20 min).

The main functions of a cardiologist during the meeting are making the plan of diagnostic tests; determining the plan and goal of the treatment; and monitoring achieved goals; if necessary, referring the patient to another specialist; adjusting medical treatment; reassessing clinical status.

The functions of the nurse in this program are broader and more time is allocated: (1) assessment of the patient (approximately 20 min); (2) patient education (approximately 45 min); (3) other functions (15–30 min).

### 2.2 Clinical data

Demographic and clinical data of the patients were collected, including information such as sex, age, New York Heart Association (NYHA) class, and comorbidities such as Type 2 diabetes mellitus (T2DM), stroke, dyslipidemia, anemia, chronic kidney disease (CKD), arterial hypertension (AH), previous myocardial infarction (MI), ischemic heart disease (IHD), significant coronary artery disease (CAD) (defined as ≥50% narrowing of the diameter of the lumen of the left main coronary artery or ≥70% narrowing of the diameter of the lumen of the left anterior descending coronary artery, left circumflex artery, or right coronary artery), atrial fibrillation (AF) were collected during the first visit, vital parameters including heart rate (HR), blood pressure (BP), waist circumference (WC) (considering WC ≥ 88 cm for women and ≥102 cm for men as enlarged), obesity grade [based on body mass index (BMI) ≥ 30], existing ankle edema (graded on a scale from 1+ (mild) to 4+ (severe)), 6-Minute Walk Test (6 MWT) dyspnea, and laboratory tests such as N-terminal pro B-type natriuretic peptide (NT-proBNP), hemoglobin, urea, potassium, sodium, uric acid, creatinine, glomerular filtration rate (GFR), aspartate aminotransferase (AST), alanine transaminase (ALT), gamma-glutamyl transferase (GGT), bilirubin, direct bilirubin, alkaline phosphatase were collected during all visits. In review echocardiographic parameters (data collected during the first and fourth visit) were also assessed to evaluate left ventricular (LV) and right ventricular systolic function.

### 2.3 Minnesota living with heart failure questionnaire (MLHFQ)

The Minnesota and European Heart Failure self-care questionnaires were used to evaluate how HF symptoms changed participants' quality of life and were assessed during scheduled visits. Other factors (symptoms, signs, and laboratory indicators of heart failure) influencing the evaluation of the monitoring effectiveness were also assessed. The MLHFQ stands as the frequently utilized tool for assessing the quality of life in patients with heart failure (8–10). The MLHFQ comprises 21 questions designed to assess the impact of heart failure on the physical, psychological, and socioeconomic aspects of patients (11). It offers a personalized perspective on various restrictive circumstances linked to the syndrome. The questions cover areas such as the signs and symptoms of heart failure, social relationships, physical and sexual activity, work, and emotions. Each question's response is selected on a scale ranging from 0 (none) to 5 (very much), with higher scores indicating a poorer quality of life (12).

### 2.4 European HF self-care questionnaire

Within this study, the self-care practices of heart failure (HF) patients were evaluated utilizing the European HF Self-Care Questionnaire (4). This questionnaire consists of 12 questions, and responses are rated on a 5-point Likert scale: “completely agree” (1 point), “agree” (2 points), “neither agree nor disagree” (3 points), “do not agree” (4 points), and “completely disagree” (5 points). The cumulative score derived from the questionnaire signifies the degree of self-care in HF patients, with a lower score indicating more effective self-care practices. The scoring categories were defined as follows: 12–27 points indicate good self-care, 28–43 points indicate satisfactory self-care, and 44–60 points indicate poor self-care.

### 2.5 Statistical analysis

Data were analyzed using the Statistical Package for the Social Sciences 27 (SPSS 27) software package (13). Continuous variables are expressed as mean ± standard deviation (SD) and were assessed using the unpaired Student's *t*-test. Categorical variables are presented as absolute numbers (percentages) and were compared using the *χ*^2^ test. Statistical significance was set as statistically significant data.

### 2.6 Ethical considerations

All participants received written information on the study aims and purpose, were informed that the confidentiality of their data was guaranteed and that they could withdraw at any time, and provided written informed consent. All data collected from the participants were identified using anonymous codes. This study was reviewed and approved by the Kaunas Regional Biomedical Research Ethics Committee (2022-10-11; Nr. P1-BE-2-5/2018).

## 3 Results

### 3.1 Clinical characteristics of the study population

The study included 118 patients experiencing deteriorating HF, categorized as I50 according to the International Classification of Diseases (ICD-10), encompassing both chronic decompensated and *de novo* cases. In Group I, the average age was 63.6 (±11.82) years, and in Group II, it was 59.06 (±13.9) years (*p* = 0.05). 61 (85.9%) patients in Group I and 40 (85.1%) in Group II were male (*p* = 0.902).

During the study, no significant differences were observed between the groups in terms of NYHA functional class (*p* = 0.182) and concurrent conditions, such as type 2 diabetes (*p* = 0.984), arterial hypertension (*p* = 0.771), atrial fibrillation (*p* = 0.453), dyslipidemia (*p* = 0.207), and cerebrovascular stroke (*p* = 0.181). However, when assessing the origin of HF, statistically significant differences were observed between the groups. In Group II, a history of myocardial infarction was more frequent (*p* = 0.038), and there was a significantly higher occurrence of significant coronary artery disease (*p* = 0.006).

During the study, no significant differences were observed between the groups in terms of NYHA functional class (*p* = 0.182) and concurrent conditions, such as type 2 diabetes (*p* = 0.984), arterial hypertension (*p* = 0.771), atrial fibrillation (*p* = 0.453), dyslipidemia (*p* = 0.207), and cerebrovascular stroke (*p* = 0.181). However, when assessing the origin of HF, statistically significant differences were observed between the groups. In Group II, a history of myocardial infarction was more frequent (*p* = 0.038), and there was a significantly higher occurrence of significant coronary artery disease (*p* = 0.006).

When comparing the clinical data across the groups, a statistically significant manifestation of ankle edema was observed in Group II (*p* = 0.05). However, no statistically significant differences were found between the groups in terms of heart rate (*p* = 0.630), blood pressure (*p* = 0.917), waist measurements (*p* = 0.917), obesity prevalence (*p* = 0.283), presence of dyspnea (*p* = 0.157), or results of the 6-Minute Walk Test (*p* = 0.283).

Laboratory parameters indicating liver function showed a statistically significant increase among the patients in Group II. The following indicators showed an increase: specifically, AST (*p* = 0.050), ALT (*p* = 0.010) and GGT (*p* = 0.031) levels were significantly affected. However, no significant differences were observed between the groups in the remaining laboratory test results (Table 1).

Analysis of the echocardiographic data (Table 1) revealed a statistically significant difference between the groups in terms of LVEF (*p* = 0.043) and TAPSE (*p* = 0.031). There were no statistically significant differences between the groups in terms of LVEDD (*p* = 0.963) or left atrial size (*p* = 0.767).

**Table 1:** Basic characteristics and comparison of clinical, laboratory parameters and echocardiographic data between groups of the patients with heart failure after long term monitoring program.

Characteristics	Group I *N* = 71	Group II *N* = 47	*p* value
Gender (male), *n* (%)	61 (85.9)	40 (85.1)	*p* = 0.902
Age, (years) ±SD	63.6 (±11.82)	59.06 (±13.9)	*p* = 0.05
NYHA class:			
I–II functional class, *n* (%)	48 (72.7)	36 (83.7)	*p* = 0.182
III–IV functional class, *n* (%)	18 (27.3)	7 (16.3)	
Comorbidities			
Atrial fibrillation, *n* (%)	36 (50.7)	20 (42.6)	*p* = 0.453
Prior myocardial infarction, *n* (%)	33 (46.50)	31 (66.0)	*p* = 0.038
Significant coronary artery disease, *n* (%)	40 (56.3)	35 (74.5)	*p* = 0.045
Type 2 diabetes mellitus, *n* (%)	15 (21.1)	10 (21.3)	*p* = 0.984
Arterial hypertension, *n* (%)	59 (83.1)	40 (85.1)	*p* = 0.771
Dyslipidemia, *n* (%)	45 (63.4)	35 (74.5)	*p* = 0.207
Cerebrovascular stroke, *n* (%)	8 (11.3)	2 (4.3)	*p* = 0.181
Clinical parameters			
Heart Rate, (bpm) (±SD)	72 (±13.18)	73 (±16.57)	*p* = 0.630
Blood Pressure, (mmHg) (±SD)	130.92 (±14.14)	131.26 (±17.98)	*p* = 0.917
Waist (>102 cm male and >88 female), *n* (%)	41 (54.7%)	28 (68.3%)	*p* = 0.917
Existing ankle oedema, *n* (%)	21 (29.5%)	16 (34.04%)	*p* = 0.05
Obesity (yes), *n* (%)	32 (42.1%)	22 (52.4%)	*p* = 0.283
Dyspnea (yes), *n* (%)	36 (48.6%)	25 (62.5%)	*p* = 0.157
6-Minute Walk Test, m (±SD)	365.90 (±115.06)	400 (±96.73)	*p* = 0.122
Laboratory parameters			
NT-proBNP, ng/L (±SD)	1,058.25 (±383.72)	403.28 (±311.89)	*p* = 0.062*
Hb, g/L (±SD)	134.48 (±16.39)	129.75 (±37.6)	*p* = 0.565
Urea, mmol/L (±SD)	8.00 (±3.73)	8.30 (±3.49)	*p* = 0.779
K, mmol/L (±SD)	4.51 (±0.52)	4.49 (±0.57)	*p* = 0.839
Na, mmol/L (±SD)	134.44 (±3.22)	136.35 (±3.57)	*p* = 0.105
Uric acid, mg/dl (±SD)	459.32 (±153.13)	435.47 (±113.77)	*p* = 0.406
Creatinine, µmol/L (±SD)	107.69 (±30.91)	111.08 (±26.77)	*p* = 0.584
(GFR), ml/min (±SD)	66.29 (±20.82)	64.13 (±24.58)	*p* = 0.677
AST, U/L (±SD)	23.06 (±9.24)	27.11 (±14.25)	*p* = 0.050
ALT, IU/L (±SD)	25.20 (±13.60)	42.21 (±53.54)	*p* = 0.010
GGT, U/L (±SD)	42.95 (±43.15)	70.63 (±84.24)	*p* = 0.031
Bilirubin, µmol/L (±SD)	17.79 (±9.27)	19.27 (±9.55)	*p* = 0.380
Direct bilirubin, µmol/L (±SD)	3.46 (±3.30)	2.80 (±1.87)	*p* = 0.306
Alkaline phosphatase, U/L (±SD)	74.80 (±19.94)	78.60 (±18.98)	*p* = 0.439
Echocardiographic data			
LVEDD, mm (±SD)	61.47 (±9.02)	61.38 (±8.90)	*p* = 0.963
LVEF, (%)	24.72 (±10.72)	29.01 (±11.66)	*p* = 0.043
Left atrial size, mm (±SD)	53.57 (±16.98)	54.65 (±14.20)	*p* = 0.767
TAPSE	13.06 (±3.53)	15.70 (±5.28)	*p* = 0.031

NYHA, New York Heart Association; NT-proBNP, N-terminal pro b-type natriuretic peptide; HB, hemoglobin; K, potassium; Na, natrium; GFR, glomerular filtration rate; AST, aspartate aminotransferase; ALT, alanine aminotransferase; GGT, gamma-glutamyl transpeptidase; LVEDD, left ventricular end-diastolic; LVEF, left ventricular ejection fraction; TAPSE, tricuspid annular plane systolic excursion.

*Data are shown as the mean ± standard deviation (SD) and non-parametric Wilcoxon test.

In addition, we analyzed changes in HRQoL dimensions during the long-term follow-up program and observed statistically significant between-group differences in the overall change in the MLHFQ (*p* < 0.001), as well as in all dimensions, including emotional, physical, and social aspects (*p* < 0.001). Comparison of HRQoL dimensions changes was defined whether V1-V4 (Table 2).

**Table 2:** Comparison of HRQoL dimensions changes during long-term monitoring program of patients (based on Minnesota living with heart failure questionnaire) in patients with heart failure.

Dimensions	Group I *N* = 71	Group II *N* = 47	*p* value
	Mean (SD)	Mean (SD)	
Emotional	6.38 (±5.2)	−1.4 (±5.9)	*p* < 0.001
Physical	12.88 (±8.1)	−3.21 (±8.33)	*p* < 0.001
Social	8.52 (±7.2)	−3.4 (±9.67)	*p* < 0.001
Total	28.82 (±16.13)	−6.91 (±19.44)	*p* < 0.001

For more detailed analysis based on the ROC (Receiver Operating Characteristic) test, we obtained the following threshold values: KSIF 17.5% (area under the ROC curve 61.1%), ALT value 27.6 (area under the ROC curve 63.3%), and age threshold value of 60 years (area under the ROC curve 59.7%) (Table 3).

**Table 3:** Characteristics and comparison of threshold values age, LVEF, significant coronary disease and ALT between groups of the patients with heart failure after long term monitoring program.

Characteristics	Group I *N* = 71	Group II *N* = 47	*p* value
Age, years			
>60 years, *n* (%)	45 (63.4)	20 (42.6)	*p* = 0.026
<60 years, *n* (%)	26 (36.6)	27 (57.4)	
LVEF, %			
>17.5%	47 (66.2)	40 (80)	*p* = 0.012
<17.5%	24 (33.8)	6 (13)	
Significant coronary artery disease, *n* (%)			
Yes (%)	40 (56.3)	35 (74.5)	*p* = 0.045
ALT, IU/L			
<27.6 *n* (%)	48 (67.1)	19 (40.9)	*p* = 0.006
>27.6 *n* (%)	23 (32.9)	28 (59.1)	

LVEF, left ventricular ejection fraction; ALT, alanine aminotransferase.

Age groups (<60 and >60 years) were directly and significantly correlated with the presence of significant coronary artery disease (yes-no) (*r* = 0.209, *p* = 0.018); conversely, they were significantly inversely correlated with the ALT group (<27.6 and >27.6) (*r* = −0.266, *p* = 0.003).

A multivariate logistic regression analysis was conducted to explore potential factors associated with a decline in quality of life. It was found that the presence of significant coronary artery disease (*p* = 0.031), reduced LVEF (*p* = 0.008), and elevated ALT (*p* = 0.001) levels are associated with worse quality of life (Tables 4, 5).

**Table 4:** Results of univariate logistic regression analysis assessing to examine the relations of the age, LVEF, significant coronary artery disease and ALT with group II during follow-up.

Characteristic	OR [95% CI]	*p* value
Age >60	2.337 [1.1–4.963]	*p* = 0.027
Significant coronary artery disease	2.26 [1.009–5.062]	*p* = 0.047
LVEF <17.5%	3.404 [1.266–9.152]	*p* = 0.015
ALT >27.6	2.952 [1.352–6.445]	*p* = 0.007

OR, odds ratio; CI, confidence interval; LVEF, left ventricular ejection fraction; ALT, alanine aminotransferase.

**Table 5:** Results of multivariate logistic regression analysis assessing to examine the relations of LVEF, significant coronary artery disease and ALT with group II during follow-up.

Characteristics	OR [95% CI]	*p* value
Significant coronary artery disease	2.697 [1.093–6.652]	*p* = 0.031
LVEF < 17.5%	4.452 [1.485–13.345]	*p* = 0.008
ALT > 27.6	4.137 [1.726–9.916]	*p* = 0.001
Constant	−0.987	*p* = 0.016

OR, odds ratio; CI, confidence interval; LVEF, left ventricular ejection fraction; ALT, alanine aminotransferase.

Although there was no statistically significant difference between the groups in the assessment of the European HF Self-Care Behavior Scale (Table 6), a statistically significant difference was found between the first and fourth visits in the assessment of the European HF Self-Care Behavior Scale (*p* = 0.0001) in both groups.

**Table 6:** Comparison of self-care changes during long-term monitoring program of patients based on the European Heart Failure Self-Care Behavior Scale in patients with heart failure.

Self-care points	Group I *N* = 71	Group II *N* = 47	*p* value
	Mean (SD)	Mean (SD)	
Visit 1^a^	28.27 (±7.47)	27.63 (±8.55)	*p* = 0.683
Visit 4^b^	22.89 (±6.71)	21.80 (±6.21)	*p* = 0.391

^a^ Cronbach alpha Visit 1—0.75.

^b^ Cronbach alpha Visit 4—0.82.

**Figure 1:**
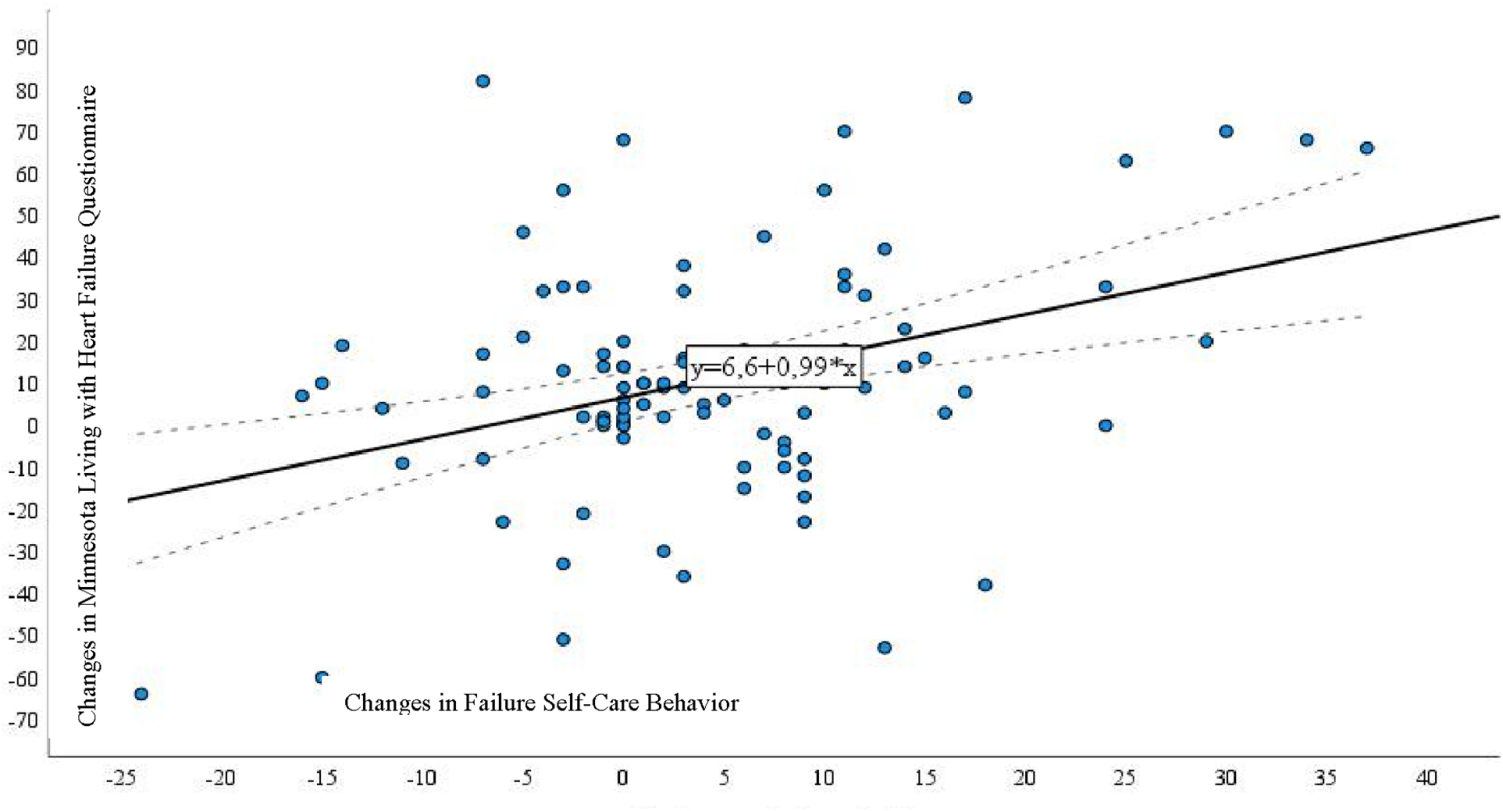
Correlation between the Minnesota Living with Heart Failure Questionnaire and European Heart Failure Self-Care Behavior Scale in patients with heart failure undergoing a long-term monitoring program.

When examining the correlation between the MLHFQ and the European HF Self-Care Behavior Scale (Figure 1), we identified a direct and statistically significant correlation between changes in self-care and MLHFQ scores (*r* = 0.221, *p* = 0.028).

## 4 Discussion

Quality of life assessment encompasses the use of generic and specific questionnaires. Notably, within the realm of specific assessments, the MLHFQ, pioneered by Rector, is the preeminent choice (14). Our results revealed that patients with an unchanged or increased MLHFQ score of less than 10 points at the fourth visit exhibited a statistically significant history of previous myocardial infarction (MI) and significant coronary artery disease (CAD).

Edema in the extremities among patients with heart failure (HF) can result from multiple factors, such as insufficient function in the left and/or right sides of the heart, autoregulatory mechanisms within the cardiovascular system, concurrent fluid retention, or even of the use of medications for treatment (15, 16). In a study by Kedler et al., which emphasized the role of physical examinations and supplementary tests in HF diagnosis within primary healthcare, a substantial occurrence of bilateral ankle edema was noted among recently diagnosed HF patients in comparison to the patients without heart failure (40.6% vs. 22%) (17). Analysis of the data from our study revealed a statistically significant occurrence of ankle edema among patients in Group II (*p* = 0.05).

Liver dysfunction is common among patients with chronic heart failure (CHF) (18). Reduced cardiac output causing hypoperfusion and congestion due to volume and pressure overload can result in hepatic injury (19). Although liver dysfunction is prevalent in heart failure (HF) patients, the prognostic significance of liver function tests (LFTs) remains a subject of debate (20). Decreased perfusion from low cardiac output and arterial hypoperfusion leads to “acute cardiogenic liver injury (ACLI),” characterized by elevated AST and ALT levels in heart failure, indicating hepatocellular damage (21). A retrospective analysis of the PARADIGM-HF trial revealed that elevated levels of alanine aminotransferase (ALT) were linked to a poorer prognosis in patients with chronic heart failure with reduced ejection fraction (HFrEF), as well as total bilirubin, while aspartate aminotransferase (AST) did not show a significant association with prognosis (22). In our study, there was a statistically significant difference between the groups in terms of specific indicators of liver function: AST (*P* = 0.050), ALT (*P* = 0.010), and GGT (*P* = 0.031). These indicators were worse among the patients in Group II.

The left ventricular ejection fraction (LVEF) is a fundamental measure of left ventricular systolic function. LVEF represents the fraction of chamber volume ejected during systole (stroke volume) relative to the volume of blood in the ventricle at the end of diastole (end-diastolic volume) (23). LVEF is the most widely utilized parameter to assess patients with heart failure (HF) (24). In the study by Wu S at all. low LVEF was associated with higher mortality and major adverse cardiovascular events (MACE) compared with normal and high LVEF (*p* < 0.0001) in women (25). By contextualizing survival within the framework of actuarial prognostication, in the study Drozd M at al. has elucidated that individuals afflicted with heart failure and diminished left ventricular ejection fraction (LVEF) exhibit a 2.4-fold increase in mortality beyond anticipated levels. The preponderance of this decrement in life expectancy is attributed to patients with comorbidity, with a pronounced impact observed among the female population (26). Ravera A et al. found in their study that although women with HFrEF had similar physician-rated symptoms, they reported worse quality of life than men (27). Some studies suggest that the quality of life (QoL) for HF patients is not dependent on the LVEF (24) and remains comparable between patients with preserved LVEF (HFpEF) and reduced LVEF (HFrEF) in contemporary randomized clinical trials, adjusting for variations in demographics, functional status, and symptom burden (28). Nevertheless, other research studies indicate notable distinctions in both total MLHFQ scores and MLHFQ subscale scores among the three groups categorized by left ventricular ejection fraction (LVEF): preserved LVEF (>50%), mid-range LVEF (40%–49%), and reduced LVEF (<40%) (*p* < 0.05). Additionally, it was observed that within the groups with lower MLHFQ scores, the HFmrEF group exhibited significantly higher rates of mortality and heart failure-related hospitalization compared to the HFpEF group (*p* = 0.035) (29).

In the present study, the analysis of electrocardiographic data revealed a statistically significant difference between the groups according to LVEF (*p* = 0.043) and TAPSE (*p* = 0.031).

The assessment of HRQoL in HF patients is crucial as it serves as a significant outcome measure, reflecting the influence of HF on their everyday existence (30, 31). Instruments for evaluating HRQoL offer a means to delve into patients' perspectives on how HF affects their daily life and overall well-being, providing insights that clinical measurements alone cannot directly capture (32). Therefore, improving HRQoL is an important treatment goal (33, 34). Research confirms that the results from the present prospective study, involving a substantial cohort of patients hospitalized for HF at various medical centers, validate and establish the reliability of the MLHFQ. Crucially, the study affirms the unidimensionality of the MLHFQ total score (35). We analyzed the changes in quality-of-life dimensions during the long-term follow-up program and observed statistically significant between group differences in the overall change in the MLHFQ (*p* < 0.001), as well as in all dimensions, including emotional, physical, and social aspects (*p* < 0.001). Despite variations in intensity, content, and personnel overseeing the implementation, self-management interventions for patients with heart failure consistently lead to enhanced outcomes directly linked to their condition. These interventions also show associations with improvements in HRQoL (36). The proficiency of patient self-management skills is one of the key aspects of heart failure (HF) management. Evidence indicates that individuals diagnosed with congestive heart failure (CHF) who exhibit elevated levels of self-care demonstrate diminished mortality rates and decreased incidences of hospital readmissions attributable to disease exacerbations (37). In examining the connections between quality of life and self-care, studies have demonstrated that reduced self-care is linked to a decline in overall HRQoL, including both physical and emotional subcomponents. These associations remained strong, with minimal influence from established covariates, and were consistent for both overall self-care and consultation for the heart failure symptom subscale (12). In our study, a statistically significant correlation was observed between alterations in self-care scores and variations in the MLHFQ score (*r* = 0.221, *p* = 0.028).

During the research, examining the factors that worsen the quality of life, we found that significant coronary artery disease (*p* = 0.031), reduced LVEF (*p* = 0.008), and elevated ALT (*p* = 0.001) levels are associated with worse quality of life. This is supported by a study conducted by K. A. Shonafi et al.

### 4.1 Limitations

The study was conducted in one of the largest Lithuanian hospitals, where patients from all over Lithuania are referred. Although, the geographical inequalities and limited access to services may prevent patients from rural areas to get consultations. Also, in the study, we did not assess the level of education of the patients, which could have an impact on the ability of the patients to absorb the teaching topics and have better self-care. Marital status was also not assessed. This aspect is also significant in assessing patients' self-care. The absence of a control group also serves as a limitation.

## 5 Conclusions

Patients who exhibited a higher left ventricular ejection fraction (LVEF) at baseline, as well as those with an etiology of ischemic heart disease (IHD), better liver function, and fewer manifestations of edema, demonstrated a statistically significant improvement in their quality of life throughout the course of the patient monitoring program. Significantly, patients whose scores on the MLHFQ decreased by >10 points demonstrated considerable enhancement in their quality of life throughout the monitoring program.

## Data Availability

The raw data supporting the conclusions of this article will be made available by the authors, without undue reservation.

## References

[1] Hajouli S Ludhwani D. Heart failure and ejection fraction. In: StatPearls. Treasure Island (FL): StatPearls Publishing (2022). PMID: 31971755. 31971755

[2] Health Information Center of the Public Health Institute. Number of Registered Diseases by Diagnostic Groups. Lithuania, Vilnius: Public Health Institute (2021). Available online at: https://stat.hi.lt/default.aspx?report_id=169 (accessed November 21, 2021).

[3] McDonagh TA Metra M Adamo M Gardner RS Baumbach A Böhm M 2021 ESC guidelines for the diagnosis and treatment of acute and chronic heart failure. Eur Heart J. (2021) 42(36):3599–726. 10.1093/eurheartj/ehab368 34447992

[4] Jaarsma T Hill L Bayes-Genis A La Rocca HB Castiello T Čelutkienė J Self-care of heart failure patients: practical management recommendations from the Heart Failure Association of the European Society of Cardiology. Eur J Heart Fail. (2021) 23(1):157–74. 10.1002/ejhf.2008 32945600 PMC8048442

[5] Choi EY Park JS Min D Lee HS Ahn JA. Association between self-management behaviour and quality of life in people with heart failure: a retrospective study. BMC Cardiovasc Disord. (2022) 22(1):90. 10.1186/s12872-022-02535-7 35260090 PMC8903718

[6] Barkhordari-Sharifabad M Saberinejad K Nasiriani K. The effect of health literacy promotion through virtual education on the self-care behaviors in patients with heart failure: a clinical trial. J Health Literacy. (2021) 6(1):51–60. 10.22038/JHL.2021.56956.1159

[7] Savarese G Lund LH. Global public health burden of heart failure. Cardiac Fail Rev. (2017) 3(1):7. 10.15420/cfr.2016:25:2 PMC5494150 28785469

[8] Sheikh Sharafi H Seyed Amini B. Assessment of health literacy and self-care in heart failure patients. J Health Literacy. (2017) 1(4):203–19. 10.22038/jhl.2017.10854

[9] Yang P He J. Chinese Herbal medicines and conventional chronic heart failure treatment for the management of chronic heart failure complicated with depression: a systematic review and meta-analysis. Evid Based Complement Alternat Med. (2020) 2020:8627928. 10.1155/2020/8627928 32382309 PMC7193286

[10] Hu Y Jiang J Xu L Wang C Wang P Yang B Symptom clusters and quality of life among patients with chronic heart failure: a cross-sectional study. Jpn J Nurs Sci. (2021) 18(1):e12366. 10.1111/jjns.12366 32857469

[11] Jonkman NH Westland H Groenwold RH Ågren S Atienza F Blue L Do self-management interventions work in patients with heart failure? An individual patient data meta-analysis. Circulation. (2016) 133(12):1189–98. 10.1161/CIRCULATIONAHA.115.018006 26873943 PMC5180429

[12] Bilbao A Escobar A García-Perez L Navarro G Quirós R. The Minnesota living with heart failure questionnaire: comparison of different factor structures. Health Qual Life Outcomes. (2016) 14:23. 10.1186/s12955-016-0425-7 26887590 PMC4756518

[13] IBM. IBM SPSS Statistics for Windows (version 27.0) [computer software]. Armonk, NY: IBM Corp (2021).

[14] Gecaite-Stonciene J Burkauskas J Bunevicius A Steibliene V Macijauskiene J Brozaitiene J Validation and psychometric properties of the Minnesota living with heart failure questionnaire in individuals with coronary artery disease in Lithuania. Front Psychol. (2022) 12:771095. 10.3389/fpsyg.2021.771095 35185680 PMC8855069

[15] Iqbal J Francis L Reid J Murray S Denvir M. Quality of life in patients with chronic heart failure and their carers: a 3-year follow-up study assessing hospitalization and mortality. Eur J Heart Fail. (2010) 12(9):1002–8. 10.1093/eurjhf/hfq114 20615920

[16] Schellong SM Wollina U Unger L Machetanz J Stelzner C. Das geschwollene bein [leg swelling]. Internist (Berl). (2013) 54(11):1294–303. 10.1007/s00108-013-3339-z 24264570

[17] Philipson H Ekman I Forslund HB Swedberg K Schaufelberger M. Salt and fluid restriction is effective in patients with chronic heart failure. Eur J Heart Fail. (2013) 15(11):1304–10. 10.1093/eurjhf/hft097 23787719

[18] Kelder JC Cramer MJ van Wijngaarden J van Tooren R Mosterd A Moons KG The diagnostic value of physical examination and additional testing in primary care patients with suspected heart failure. Circulation. (2011) 124(25):2865–73. 10.1161/CIRCULATIONAHA.111.019216 22104551

[19] Poelzl G Ess M Mussner-Seeber C Pachinger O Frick M Ulmer H. Liver dysfunction in chronic heart failure: prevalence, characteristics and prognostic significance. Eur J Clin Invest. (2012) 42(2):153–63. 10.1111/j.1365-2362.2011.02573.x 21806605

[20] Samsky MD Patel CB DeWald TA Smith AD Felker GM Rogers JG Cardiohepatic interactions in heart failure: an overview and clinical implications. J Am Coll Cardiol. (2013) 61(24):2397–405. 10.1016/j.jacc.2013.03.042 23603231

[21] Liang W He X Wu D Xue R Dong B Owusu-Agyeman M Prognostic implication of liver function tests in heart failure with preserved ejection fraction without chronic hepatic diseases: insight from TOPCAT trial. Front Cardiovasc Med. (2021) 8:618816. 10.3389/fcvm.2021.618816 34055924 PMC8153182

[22] Suzuki K Claggett B Minamisawa M Packer M Zile MR Rouleau J Liver function and prognosis, and influence of sacubitril/valsartan in patients with heart failure with reduced ejection fraction. Eur J Heart Fail. (2020) 22:1662–71. 10.1002/ejhf.1853 32407608

[23] Correale M Tarantino N Petrucci R Tricarico L Laonigro I Di Biase M Liver disease and heart failure: back and forth. Eur J Intern Med. (2018) 48:25–34. 10.1016/j.ejim.2017.10.016 29100896

[24] Kosaraju A Goyal A Grigorova Y Makaryus AN. Left ventricular ejection fraction. In: StatPearls. Treasure Island (FL): StatPearls Publishing (2023). PMID: 29083812. 29083812

[25] Wu S Wei J Lauzon M Suppogu N Kelsey SF Reis SE Left ventricular ejection fraction and long-term outcomes in women presenting with signs and symptoms of ischemia. Heart (Br Cardiac Soc). (2023) 109(21):1624–30. 10.1136/heartjnl-2023-322494 PMC10592103 37316162

[26] Drozd M Relton SD Walker AMN Slater TA Gierula J Paton MF Association of heart failure and its comorbidities with loss of life expectancy. Heart (Br Cardiac Soc). (2021) 107(17):1417–21. 10.1136/heartjnl-2020-317833 PMC8372397 33153996

[27] Ravera A Santema BT Sama IE Meyer S Lombardi CM Carubelli V Quality of life in men and women with heart failure: association with outcome, and comparison between the Kansas city cardiomyopathy questionnaire and the EuroQol 5 dimensions questionnaire. Eur J Heart Fail. (2021) 23(4):567–77. 10.1002/ejhf.2154 33728762 PMC8252457

[28] Mele D Nardozza M Ferrari R. Left ventricular ejection fraction and heart failure: an indissoluble marriage? Eur J Heart Fail. (2018) 20(3):427–30. 10.1002/ejhf.1071 29314500

[29] Chandra A Vaduganathan M Lewis EF Claggett BL Rizkala AR Wang W PARAGON-HF investigators health-related quality of life in heart failure with preserved ejection fraction: the PARAGON-HF trial. JACC Heart Fail. (2019) 7(10):862–74. 10.1016/j.jchf.2019.05.015 31302043

[30] Chen X Xin Y Hu W Zhao Y Zhang Z Zhou Y. Quality of life and outcomes in heart failure patients with ejection fractions in different ranges. PLoS One. (2019) 14(6):e0218983. 10.1371/journal.pone.0218983 31247042 PMC6597164

[31] Heo S Moser DK Riegel B Hall LA Christman N. Testing the psychometric properties of the Minnesota living with heart failure questionnaire. Nurs Res. (2005) 54(4):265–72. 10.1097/00006199-200507000-00009 16027569

[32] Westlake C Dracup K Creaser J Livingston N Heywood JT Huiskes BL Correlates of health-related quality of life in patients with heart failure. Heart Lung. (2002) 31(2):85–93. 10.1067/mhl.2002.122839 11910383

[33] Garin O Soriano N Ribera A Ferrer M Pont A Alonso J Validación de la versión española del Minnesota living with heart failure questionnaire [Validation of the Spanish version of the Minnesota living with heart failure questionnaire]. Rev Esp Cardiol. (2008) 61(3):251–9. 10.1157/13116652 18361898

[34] Moon JR Jung YY Jeon ES Choi JO Hwang JM Lee SC. Reliability and validity of the Korean version of the Minnesota living with heart failure questionnaire. Heart Lung. (2012) 41(1):57–66. 10.1016/j.hrtlng.2011.09.011 22195494

[35] Alla F Briançon S Guillemin F Juillière Y Mertès PM Villemot JP Self-rating of quality of life provides additional prognostic information in heart failure. Insights into the EPICAL study. Eur J Heart Fail. (2002) 4(3):337–43. 10.1016/s1388-9842(02)00006-5 12034160

[36] Jenča D Melenovský V Stehlik J Staněk V Kettner J Kautzner J Heart failure after myocardial infarction: incidence and predictors. ESC Heart Fail. (2021) 8(1):222–37. 10.1002/ehf2.13144 33319509 PMC7835562

[37] Wiśnicka A Lomper K Uchmanowicz I. Self-care and quality of life among men with chronic heart failure. Front Public Health. (2022) 10:942305. 10.3389/fpubh.2022.942305 35937256 PMC9354614

